# Three-Dimensional Tracking of a Target under Angle-Frequency Measurements with Multiple Frequency Lines

**DOI:** 10.3390/s23125705

**Published:** 2023-06-19

**Authors:** Jonghoek Kim

**Affiliations:** System Engineering Department, Sejong University, Seoul 05006, Republic of Korea; jonghoek@gmail.com

**Keywords:** underwater target tracking, multiple frequency lines, angle-frequency target motion analysis, 3D AFTMA

## Abstract

This article considers tracking a constant-velocity underwater target, which emits sound with distinct frequency lines. By analyzing the target’s azimuth, elevation and multiple frequency lines, the ownship can estimate the target’s position and (constant) velocity. In our paper, this tracking problem is called the 3D Angle-Frequency Target Motion Analysis (AFTMA) problem. We consider the case where some frequency lines disappear and appear occasionally. Instead of tracking every frequency line, this paper proposes to estimate the average emitting frequency by setting the average frequency as the state vector in the filter. As the frequency measurements are averaged, the measurement noise decreases. In the case where we use the average frequency line as our filter state, both the computational load and the root mean square error (RMSE) decrease, compared to the case where we track every frequency line one by one. As far as we know, our manuscript is unique in addressing 3D AFTMA problems, such that an ownship can track an underwater target while measuring the target’s sound with multiple frequency lines. The performance of the proposed 3D AFTMA filter is demonstrated utilizing MATLAB simulations.

## 1. Introduction

This article considers a scenario where an ownship tracks a constant-velocity underwater target, which emits sound with distinct frequency lines. For instance, this scenario can be utilized for tracking of an enemy submarine by processing the sound generated from the submarine.

Multi-frequency lines, concurrently processed with bearing measurements, can be used for tracking a constant-velocity target [1]. For example, the target’s engines or propellers make noise with distinct frequency lines. Moreover, some frequency lines may disappear and appear occasionally [2].

Inspired by [1], our paper handles the case where a constant-velocity target generates multiple frequency lines. Reference [1] addressed a typical architecture for processing underwater sonar measurements, so that a target’s bearing and frequency lines can be estimated in real time.

By analyzing the target’s azimuth, elevation and multiple frequency lines, the ownship can estimate the target’s position and (constant) velocity. In our paper, this 3D tracking problem is called the 3D Angle-Frequency Target Motion Analysis (AFTMA) problem. The proposed tracking filter can handle multiple frequency lines as well as disappearance of a frequency line. As far as we know, no paper in the literature on 3D AFTMA has considered both multiple frequency lines and disappearance of a frequency line.

Considering 2D scenarios, the ownship can track a target based on bearing and frequency measurements [1,3,4,5,6,7]. Considering 2D cluttered environments, ref. [4] addressed a solution to multi-target tracking based on the Gaussian mixture probability hypothesis density (GM-PHD) filter jointly using the frequency and bearing measurements. In the case where the ownship can measure the target’s frequency and bearing, the ownship’s maneuver is not necessary for tracking a constant-velocity target, provided that the unknown emission frequency is a constant and that the bearing rate is not equal to zero [1].

Considering 3D scenarios, refs. [8,9] addressed the 3D AFTMA problem, where the ownship tracks a target based on frequency, elevation, and bearing measurements. The authors of [9] addressed the 3D AFTMA, by considering measurement parameters such as frequency, elevation and bearing of the target. In ref. [8], the Gauss–Helmert model was introduced to solve the 3D AFTMA problem by expanding the unknown signal emission time as an unknown variable to the state. The papers reviewed in this paragraph did not consider the case where a target generates multiple frequency lines. Note that this distinguishes our paper from other papers on 3D AFTMA.

Reference [1] considered 2D scenarios for tracking a constant-velocity target generating multiple frequency lines. However, ref. [1] did not consider 3D environments, as in our paper. In addition, ref. [1] did not consider the fact that a frequency line may disappear and appear occasionally. Ref. [1] considered the ideal case where all frequency lines exist at all times. However, ref. [1] cannot handle a practical scenario where a frequency line may disappear and appear occasionally.

Reference [2] considered 2D scenarios for tracking a constant-velocity target, in the case where there are two frequency lines. Reference [2] considered the case where a frequency line appears and disappears occasionally. However, ref. [2] considered 2D scenarios where only two frequency lines exist. Distinct from [1,2], our paper addresses the 3D AFTMA problem, considering the case where multiple frequency lines can disappear and appear occasionally.

Instead of tracking every frequency line one by one, we propose to estimate the average emitting frequency by setting the average frequency as the state vector in the filter. As the frequency measurements are averaged, the measurement noise decreases. Moreover, using the average frequency as our Kalman filter state, we can decrease the matrix size of the covariance matrix in the Kalman filter. This decreases the numerical error in matrix calculations, such as the calculation of an inverse matrix. Using MATLAB simulations, we show that as we use the average frequency line as our filter state, both the computational load and the root mean square error (RMSE) decrease, compared to the case where we track every frequency line one by one.

As far as we know, our paper is novel in addressing the 3D AFTMA with multiple frequency lines, such that a frequency line may disappear or appear occasionally. The performance of the proposed AFTMA filter is further demonstrated utilizing MATLAB simulations.

This paper is organized as follows. Section 2 presents the 3D AFTMA problem handled in this paper. Section 3 presents the proposed tracking filter for solving the 3D AFTMA problem with multiple frequency lines. Section 4 presents the MATLAB simulations. Section 5 presents the conclusions.

## 2. 3D AFTMA Problem

Before addressing the 3D AFTMA problem, some definitions are presented. Let $I_{a}$ denote an identity matrix with size a×a. Let $0_{a}$ denote a zero matrix with size a×a. Let $0_{a,1}$ denote a zero vector with size a×1. In addition, diag(a,b,c,…) denotes a diagonal matrix with diagonal elements a,b,c,… in this order. Let C[i] denote the *i*-th element in a column vector C. Let C[1:n] denote a column vector composed of first *n* elements in C. Considering a matrix M, M[1:n,1:n] indicates the sub-matrix of M, consisting of the first *n* rows and *n* columns of M. Let Cov(q) denote the error covariance for a variable q for convenience. Let N(0,M) indicate the Gaussian distribution with mean 0 and variance M.

This manuscript addresses discrete time systems. Let dt indicate the sampling interval in discrete time systems. At sample-step *k*, some frequency lines are measured by the ownship [1]. Suppose that $M_{k}$ frequency lines are measured at sample-step *k*. At sample-step *k*, we sort measured frequency lines in ascending order. Let $f_{k}^{e,m}$ ($m\in \{1,2,\ldots ,M_{k}\}$) denote the emitting frequency of the *m*-th frequency line, which is sorted in the ascending order. This implies that

(1) $$\begin{array}{c} f_{k}^{e,m}\leq f_{k}^{e,n} \end{array}$$

in the case where $m<n\leq M_{k}$.

Let

(2) $$\begin{array}{c} S_{k}^{e}=\left\{f_{k}^{e,1},f_{k}^{e,2},\ldots ,f_{k}^{e,M_{k}}\right\} \end{array}$$

denote the sorted emitting frequency set, composed of $f_{k}^{e,m}$ for all $m\in \{1,2,\ldots ,M_{k}\}$. Since frequency lines can appear and disappear as time goes on, $S_{k}^{e}\neq S_{k^{\prime }}^{e}$ is feasible for $k\neq k^{\prime }$.

It is assumed that the emitting frequency of a frequency line is a constant. This assumption has been widely applied in tracking problems based on frequency measurements [1,2,3,4,8,10]. For instance, suppose that an emitting frequency, say $f^{e}$, exists at all sample-steps *k*. Then, $f^{e}\in S_{k}^{e}$ for all sample-steps *k*.

For all $m\in \{1,2,\ldots ,M_{k}\}$, $f_{k}^{e,m}$ is not known a priori. Suppose that one builds a filter of estimating $S_{k}^{e}$, i.e., we wish to estimate $f_{k}^{e,m}$ for all $m\in \{1,2,\ldots ,M_{k}\}$. This implies that we build a filter for tracking every frequency line whenever a new line appears.

If $M_{k}$ increases as sample-step *k* increases, then the number of unknown variables in $S_{k}^{e}$ increases in the filter. This increases the size of the state vector in the filter. Thus, increasing $M_{k}$ increases the computational load for estimation of unknown emitting frequencies in $S_{k}^{e}$.

Instead of estimating all emitting frequencies in $S_{k}^{e}$, the proposed approach is to estimate the average emitting frequency by setting the average frequency as the state vector in the filter. In other words, we propose to estimate the average emitting frequency by setting the average frequency as the state vector in the filter.

The authors of [1] considered the ideal case where all frequency lines exist at all times. For this ideal case, we derived the following results under MATLAB simulations (see Section 4). As we track every frequency line one by one, the computational load increases, compared to the case where we use the average frequency line as our filter state. Moreover, as we track every frequency line one by one, the RMSE increases, compared to the case where we use the average frequency line as our filter state.

By setting the average frequency as the state vector in the filter, increasing $M_{k}$ does not increase the size of the state vector in the filter. This study thus defines the average emitting frequency as

(3) $$\begin{array}{c} f_{k}^{e,avg}=\frac{1}{M_{k}}\sum_{m=1}^{M_{k}}f_{k}^{e,m}. \end{array}$$

Note that $f_{k}^{e,avg}$ in (3) is not known in advance, since $f_{k}^{e,m}$ is not known a priori. Hence, one addresses a time-efficient filter for real-time estimation of $f_{k}^{e,avg}$.

Recall that the sorted emitting frequency set $S_{k}^{e}$ is defined in (2). In practice, there may be a case where $S_{k-1}^{e}\neq S_{k}^{e}$. This implies that a frequency line can appear or disappear at sample-step *k*. A frequency line change at sample-step *k* leads to a sudden jump in the average emitting frequency $f_{k}^{e,avg}$.

### 2.1. Process Model

The target’s 3D position, velocity, and emitting frequency at sample-step *k* are denoted as $X_{k}^{E}={\left(x_{k}^{E},y_{k}^{E},z_{k}^{E},{\dot{x}}_{k}^{E},{\dot{y}}_{k}^{E},{\dot{z}}_{k}^{E},f_{k}^{e,avg}\right)}^{T}$. Considering a constant velocity (constant speed and heading) target, the target’s motion model is

(4) $$\begin{array}{c} X_{k+1}^{E}=FX_{k}^{E}+n_{k}, \end{array}$$

where $n_{k}=\Gamma \times {\left(a_{x},a_{y},a_{z}\right)}^{T}$. In $n_{k}$, ${\left(a_{x},a_{y},a_{z}\right)}^{T}$ denotes a target’s acceleration that is not known in advance. In addition, Γ is

(5) $$\begin{array}{c} \Gamma =\left(\begin{array}{c} \frac{{\left(dt\right)}^{2}}{2}\times I_{3}\\ dt\times I_{3}\\ 0_{3,1}^{T} \end{array}\right). \end{array}$$

In (4), F is

(6) $$\begin{array}{c} F=\left(\begin{array}{ccc} I_{3} & dtI_{3} & 0_{3,1}\\ 0_{3} & I_{3} & 0_{3,1}\\ 0_{3,1}^{T} & 0_{3,1}^{T} & 1 \end{array}\right). \end{array}$$

In (4), $n_{k}=\Gamma \times {\left(a_{x},a_{y},a_{z}\right)}^{T}$ is distributed according to $n_{k}∼N\left(0,Q_{k}\right)$ where

(7) $$\begin{array}{c} Q_{k}=\Gamma \times diag\left(\sigma_{a_{x}}^{2},\sigma_{a_{y}}^{2},\sigma_{a_{z}}^{2}\right)\times \Gamma^{T}, \end{array}$$

in which $\sigma_{a}$ presents the standard deviation of *a*.

Our tracking filter uses the relative state vector $X_{k}$ as follows.

(8) $$\begin{array}{c} X_{k}=X_{k}^{E}-X_{k}^{O} \end{array}$$

where $X_{k}^{O}={\left(x_{k}^{O},y_{k}^{O},z_{k}^{O},{\dot{x}}_{k}^{O},{\dot{y}}_{k}^{O},{\dot{z}}_{k}^{O},0\right)}^{T}$ defines the ownship’s 3D position and velocity at sample-step *k*. Let $X_{k}={\left(x_{k},y_{k},z_{k},{\dot{x}}_{k},{\dot{y}}_{k},{\dot{z}}_{k},f_{k}^{e,avg}\right)}^{T}$ denote the state vector in our tracking filter. The relative state vector $X_{k}$ is distinct from the target state vector $X_{k}^{E}$ in (4).

Under (4) and (8), one derives

(9) $$\begin{array}{c} X_{k+1}=F\left(X_{k}+X_{k}^{O}\right)-X_{k+1}^{O}+n_{k}. \end{array}$$

### 2.2. Measurement Model

In the 3D AFTMA problem, the target’s elevation, azimuth, and frequency are measured at sample-step *k*. Suppose that the ownship measures $M_{k}$ frequency lines at sample-step *k*. Let $f_{k}^{m}$ denote the *m*-th frequency line measurement ($m\in \{1,2,\ldots ,M_{k}\}$).

The frequency measurement equation is derived as follows. Let *C* denote the underwater sound speed, which is assumed to be accessible a priori. This assumption has been widely applied in tracking problems based on frequency measurements [1,2,3,4,8,10]. Then, $f_{k}^{m}$ ($m\in \{1,2,\ldots ,M_{k}\}$) is

(10) $$\begin{array}{c} f_{k}^{m}=f_{k}^{e,m}\times \left(1-\frac{{\dot{r}}_{k}}{C}\right)+n_{f}. \end{array}$$

In (10), $n_{f}$ is the measurement noise with Gaussian distribution given as $n_{f}∼N\left(0,\sigma_{f}^{2}\right)$. We assume that the standard deviation of every frequency line is identical.

In (10), $r_{k}=∥X_{k}\left[1:3\right]∥$ denotes the range between the ownship and the target at sample-step *k*. Thus, ${\dot{r}}_{k}$ in (10) is

(11) $$\begin{array}{c} {\dot{r}}_{k}=\frac{X_{k}\left[1\right]\times X_{k}\left[4\right]+X_{k}\left[2\right]\times X_{k}\left[5\right]+X_{k}\left[3\right]\times X_{k}\left[6\right]}{∥X_{k}\left[1:3\right]∥}. \end{array}$$

The entire measurements at sample-step *k* are represented as

(12) $$\begin{array}{c} H\left(X_{k}\right)={\left[b_{1}\left(X_{k}\right),b_{2}\left(X_{k}\right),f_{k}^{1},f_{k}^{2},\ldots ,f_{k}^{M_{k}}\right]}^{T}. \end{array}$$

In (12), we use

(13) $$\begin{array}{c} b_{1}\left(X_{k}\right)=\tan^{-1}\left(\frac{y_{k}}{x_{k}}\right)+n_{b_{1}} \end{array}$$

and

(14) $$\begin{array}{c} b_{2}\left(X_{k}\right)=\tan^{-1}\left(\frac{z_{k}}{\sqrt{x_{k}^{2}+y_{k}^{2}}}\right)+n_{b_{2}}. \end{array}$$

Here, $b_{1}\left(X_{k}\right)$ and $b_{2}\left(X_{k}\right)$ define the azimuth and elevation angles, respectively. In (13), $n_{b_{1}}$ denotes the azimuth measurement noise with Gaussian distribution given as $n_{b_{1}}$∼$N(0,\sigma_{b,1}^{2})$. In (14), $n_{b_{2}}$ denotes the elevation measurement noise with Gaussian distribution given as $n_{b_{2}}$∼$N(0,\sigma_{b,2}^{2})$.

Using (12) as the measurement equation, we can build a 3D AFTMA filter for tracking every frequency line whenever a new frequency line appears. However, as the number of frequency lines increases, the state vector size of the filter increases. The authors of [1] stated that more than 100 frequency lines can exist in practice. In the case where we track every frequency line one by one, the covariance matrix of the associated Kalman filter has a size bigger than 100×100.

One can argue that recent high-end computers can handle the case where the size of the state vector is arbitrarily large. However, there may be a case where the ownship needs to track multiple targets. As we consume more power on tracking a single target, the total number of targets tracked by the ownship must decrease. Therefore, it is desirable to minimize the computational load for tracking a single target.

Moreover, 3D AFTMA has a large uncertainty in target range initially. For reducing the initial range estimate error in target-tracking problems, the RPEKF was applied [11,12]. The RPEKF uses multiple independent EKFs, each with a different initial range estimate, and the merged estimate is calculated as a weighted sum of individual EKF outputs. For addressing 3D AFTMA, we can apply multiple independent EKFs that are initialized at distinct range intervals, inspired by the RPEKF. However, as we consume more power on a single EKF track, we need to consume much more power as we use multiple EKF tracks. Therefore, it is desirable to minimize the computational load for a single EKF track.

Instead of tracking every frequency line one by one, we propose to estimate the average emitting frequency by setting the average frequency as the state vector in the filter. As the frequency measurements are averaged, the measurement noise decreases. Moreover, using the average frequency as our Kalman filter state, we can decrease the matrix size of the covariance matrix in the Kalman filter. This decreases the numerical error in matrix calculations, such as the calculation of an inverse matrix.

We show the outperformance of applying the average frequency using MATLAB simulations. As we use the average frequency line as our filter state, both the computational load and the RMSE decrease, compared to the case where we track every frequency line one by one.

At each sample-step *k*, one averages all frequency line measurements to obtain

(15) $$\begin{array}{c} f_{k}^{avg}=\frac{1}{M_{k}}\times \sum_{m=1}^{M_{k}}f_{k}^{m}. \end{array}$$

Then, (3) and (10) lead to

(16) $$\begin{array}{c} f_{k}^{avg}=f_{k}^{e,avg}\times \left(1-\frac{{\dot{r}}_{k}}{C}\right)+n^{f,avg}, \end{array}$$

where $n^{f,avg}$ has a Gaussian distribution with mean 0 and standard deviation $\sigma_{f,avg}=\frac{\sigma_{f}}{\sqrt{M_{k}}}$. The standard deviation of $n^{f,avg}$ is smaller than $\sigma_{f}$. This implies that one can reduce the effect of noise as $M_{k}$ (the number of frequency lines) increases. In other words, as the frequency measurements are averaged, the measurement noise decreases.

Considering the 3D AFTMA problem with average frequency measurements in (15), the measurement equation at sample-step *k* is

(17) $$\begin{array}{c} m_{k}=h\left(X_{k}\right)+e_{k}. \end{array}$$

In (17), $e_{k}$ is the measurement noise with Gaussian distribution given as $e_{k}∼N\left(0,R_{k}\right)$. Here, $R_{k}=diag\left(\sigma_{b,1}^{2},\sigma_{b,2}^{2},\sigma_{f,avg}^{2}\right)$. Recall that $\sigma_{f,avg}=\frac{\sigma_{f}}{\sqrt{M_{k}}}$ in (16).

Using (13) and (14), $h(X_{k})$ in (17) is calculated as

(18) $$\begin{array}{c} h\left(X_{k}\right)={\left(\tan^{-1}\left(\frac{X_{k}\left[2\right]}{X_{k}\left[1\right]}\right),\tan^{-1}\left(\frac{X_{k}\left[3\right]}{\sqrt{X_{k}{\left[1\right]}^{2}+X_{k}{\left[2\right]}^{2}}}\right),f^{a}\left(X_{k}\right)\right)}^{T}. \end{array}$$

Recall that in $X_{k}$, $X_{k}\left[7\right]=f_{k}^{e,avg}$, which appears in (3). In (18), $f^{a}\left(X_{k}\right)$ is the frequency measurement equation associated with (16) and is defined as

(19) $$\begin{array}{c} f^{a}\left(X_{k}\right)=X_{k}\left[7\right]\times \left(1-\frac{{\dot{r}}_{k}}{C}\right), \end{array}$$

where ${\dot{r}}_{k}$ is defined in (11).

## 3. Proposed Tracking Filter

We introduce the 3D AFTMA tracking filter applied in our paper. Recall that our tracking filter uses the relative state vector $X_{k}$ in (8). Let ${\hat{X}}_{k|k}$ denote the estimation of $X_{k}$ based on all measurements up to sample-step *k*. In addition, let $P_{k|k}$ denote the error covariance of ${\hat{X}}_{k|k}$. Let ${\hat{X}}_{k|k-1}$ denote the prediction of $X_{k}$, based on all measurements up to sample-step k−1. Let $P_{k|k-1}$ denote the error covariance of ${\hat{X}}_{k|k-1}$.

Under the EKF with process model (9) and measurement model (17), one updates the state vector ${\hat{X}}_{k|k}$ and its covariance $P_{k|k}$ at every sample-step *k*. The detailed process of the EKF is discussed in [13].

### 3.1. A Frequency Line Appears or Disappears at Sample-Step *k*

Instead of estimating all emitting frequencies in $S_{k}^{e}$, this study runs the EKF for estimating $f_{k}^{e,avg}$, the average of emitting frequency. One presents a gating approach to determine whether a frequency average measurement $f_{k}^{avg}$ in (16) can be applied for updating the EKF at sample-step *k*.

Recall that the sorted emitting frequency set $S_{k}^{e}$ is defined in (2). Every element in $S_{k}^{e}$ is sorted using (1). In practice, there may be a case where $S_{k-1}^{e}\neq S_{k}^{e}$. This implies that a frequency line can appear or disappear at sample-step *k*. A frequency line change at sample-step *k* leads to a sudden jump in the average emitting frequency $f_{k}^{e,avg}$.

Using all frequency measurements $f_{k}^{m}$ ($m\in \{1,2,\ldots ,M_{k}\}$) at sample-step *k*, we estimate all elements in $S_{k}^{e}$ as

(20) $$\begin{array}{c} {\hat{f}}_{k}^{e,m}=\frac{f_{k}^{m}}{1-\frac{{\dot{r}}_{k}}{C}}. \end{array}$$

where ${\dot{r}}_{k}$ appears in (11).

Similarly, using all frequency measurements $f_{k-1}^{n}$ ($n\in \{1,2,\ldots ,M_{k-1}\}$) at sample-step k−1, we estimate all elements in $S_{k-1}^{e}$ as

(21) $$\begin{array}{c} {\hat{f}}_{k-1}^{e,n}=\frac{f_{k-1}^{n}}{1-\frac{{\dot{r}}_{k-1}}{C}}. \end{array}$$

Then, two *gating conditions* are as follows.

(22) $$\begin{array}{c} M_{k}==M_{k-1} \end{array}$$

and

(23) $$\begin{array}{c} max_{m\in \{1,2,\ldots ,M_{k}\}}∥{\hat{f}}_{k}^{e,m}-{\hat{f}}_{k-1}^{e,m}∥<\beta \times \sigma_{f}. \end{array}$$

Here, max(S) denotes the maximum value of all elements in a set, say *S*. Satisfying both (22) and (23) indicates that we satisfy $S_{k-1}^{e}==S_{k}^{e}$. In (23), β is a tuning parameter. In the case where (22) or (23) is not met at sample-step *k*, $S_{k-1}^{e}\neq S_{k}^{e}$.

### 3.2. Reset the EKF Estimate for Emitting Frequency

If both (22) and (23) are satisfied at sample-step *k*, then one runs the EKF measurement update associated with $f_{k}^{avg}$. Otherwise, we reset the EKF estimate for emitting frequency, since frequency lines change at sample-step *k*.

In the case where the gating conditions are not satisfied at sample-step *k*, one can consider skipping the measurement update associated with $f_{k}^{avg}$. Skipping the measurement update is useful if the new frequency average measurement is associated with the existing frequency state $X_{k}\left[7\right]$. However, our problem is that a new frequency line can suddenly appear at sample-step *k* and that the new frequency line may not be associated with the existing frequency state $X_{k}\left[7\right]$. In the worst case, all new frequency lines appear at one sample-step, while the existing frequency lines disappear at the moment. This worst case scenario is simulated in Section 4.1.

Thus, we cannot apply skipping a measurement update in the case where the gating conditions are not satisfied at sample-step *k*. Instead, we reset the EKF estimate for emitting frequency, since frequency lines can suddenly appear at sample-step *k*.

One addresses how to reset the EKF estimate for emitting frequency, in the case where the gating conditions are not satisfied at sample-step *k*. Using (11) and (16), $f_{k}^{e,avg}$ is estimated as

(24) $$\begin{array}{c} {\hat{f}}_{k}^{e,avg}=\frac{f_{k}^{avg}}{1-\frac{\dot{r}\left({\hat{X}}_{k|k-1}\right)}{C}}. \end{array}$$

Here, $\dot{r}\left({\hat{X}}_{k|k-1}\right)$ indicates (11) when we use ${\hat{X}}_{k|k-1}$ instead of $X_{k}$. Furthermore, (24) implies that this study uses the estimate ${\hat{X}}_{k|k-1}$ for the estimation of range rate $\dot{r}$. It is acknowledged that the noise term $n^{f,avg}$ in (16) is ignored in the derivation of (24). Recall that when using (16), $n^{f,avg}$ has a Gaussian distribution with mean 0 and standard deviation $\sigma_{f,avg}=\frac{\sigma_{f}}{\sqrt{M_{k}}}$. Thus, $\sigma_{f,avg}$ decreases as $M_{k}$ increases. This implies that as we use more frequency lines, the accuracy of (24) improves.

Recall that $X_{k}={\left(x_{k},y_{k},z_{k},{\dot{x}}_{k},{\dot{y}}_{k},{\dot{z}}_{k},f_{k}^{e,avg}\right)}^{T}$ denotes the state vector in our paper. The estimate vector ${\hat{X}}_{k|k}\left[1:6\right]$ is associated with the position and velocity of the target. One resets ${\hat{X}}_{k|k}\left[1:6\right]$ using

(25) $$\begin{array}{c} {\hat{X}}_{k|k}\left[1:6\right]={\hat{X}}_{k|k-1}\left[1:6\right]. \end{array}$$

We further reset ${\hat{X}}_{k|k}\left[7\right]$ (EKF estimate for emitting frequency) utilizing ${\hat{f}}_{k}^{e,avg}$ in (24). This implies that

(26) $$\begin{array}{c} {\hat{X}}_{k|k}\left[7\right]={\hat{f}}_{k}^{e,avg}. \end{array}$$

The error covariance $P_{k|k}\left[1:6,1:6\right]$ is associated with the position and velocity of the target. We use

(27) $$\begin{array}{c} Cov\left({\hat{X}}_{k|k}\left[1:6\right]\right)=Cov\left({\hat{X}}_{k|k-1}\left[1:6\right]\right). \end{array}$$

However, the error covariance for $X_{k}\left[7\right]$ needs to be reset. Using (24), one resets $Cov(X_{k}\left[7\right])$ as

(28) $$\begin{array}{c} Cov\left(X_{k}\left[7\right]\right)={\left(\frac{\sigma_{f,avg}}{1-\frac{V_{max}}{C}}\right)}^{2}. \end{array}$$

Here, $\sigma_{f,avg}=\frac{\sigma_{f}}{\sqrt{M_{k}}}$ using (16). In addition, $V_{max}$ is the target’s maximum speed that is assumed to be accessible in advance.

In summary, $P_{k|k}$ is reset utilizing

(29) $$\begin{array}{c} P_{k|k}=\left(\begin{array}{cc} P_{k|k-1}\left[1:6,1:6\right] & 0_{6,1}\\ 0_{1,6} & Cov(X_{k}\left[7\right]) \end{array}\right). \end{array}$$

## 4. MATLAB Simulations

MATLAB simulations are presented to verify the effectiveness of the proposed 3D AFTMA filter. A simulation scenario lasts for 200 s. The sampling duration is set as dt=0.1 s. According to [1], the sound speed in underwater environments is set as C=1500 m/s.

Let $\sigma_{a}$ define the standard deviation of *a*. In (7), one uses $\sigma_{a_{x}}=\sigma_{a_{y}}=\sigma_{a_{z}}=0.1$ (m/s${}^{2}$). In (17), the angle noise ($\sigma_{b,1}$ or $\sigma_{b,2}$) is set as 1 degree. In addition, the frequency noise $\sigma_{f}$ is set as 0.5 Hz. These noise parameters are feasible according to [1,2]. In a gating condition (23), we apply β=100.

Let $d_{0}$ denote the relative distance between the ownship and the target at sample-step 0. Let $d_{e}=$ 10,000 (m) denote the range estimate error at sample-step 0. In the EKF, ${\hat{X}}_{0|0}$ is initialized as

(30) $$\begin{array}{c} {\hat{X}}_{0|0}=\left(\begin{array}{c} \left(d_{0}+d_{e}\right)\times c\left(b_{2,0}\right)\times c\left(b_{1,0}\right)\\ \left(d_{0}+d_{e}\right)\times c\left(b_{2,0}\right)\times s\left(b_{1,0}\right)\\ \left(d_{0}+d_{e}\right)\times s\left(b_{2,0}\right)\\ 0_{3,1}\\ f_{0}^{avg} \end{array}\right). \end{array}$$

Here, $b_{1,0}$ and $b_{2,0}$ denote the azimuth and the elevation at sample-step 0, respectively. In addition, $f_{0}^{avg}$ denotes the average frequency measurement at sample-step 0. See (16) for the equation of $f_{k}^{avg}$.

The error covariance $P_{0|0}$ is set as

(31) $$\begin{array}{c} P_{0|0}=\left(\begin{array}{ccc} Cov({\hat{X}}_{0|0}\left(1:3\right)) & 0_{3} & 0_{3,1}\\ 0_{3} & I_{3}\times V_{max}^{2}/3 & 0_{3,1}\\ 0_{3,1}^{T} & 0_{3,1}^{T} & {\left(\delta f\right)}^{2}/3 \end{array}\right). \end{array}$$

Here,

(32) $$\begin{array}{c} Cov\left({\hat{X}}_{0|0}\left(1:3\right)\right)=diag\left(\frac{d_{e}^{2}}{3},\frac{d_{e}^{2}}{3},\frac{d_{e}^{2}}{3}\right). \end{array}$$

As the maximum speed of the target, this study utilizes $V_{max}=100$ m/s. Under (19), one utilizes $\delta f=\frac{f_{0}^{avg}\times V_{max}}{C}$ in (31).

For robust verification of the proposed tracking filter, one runs $M_{c}=100$ Monte-Carlo (MC) simulations per each scenario. Let $E_{k}^{j}\in R^{3}$, where $j\in \{1,2,,,M_{c}\}$ denote the target position estimation at sample-step *k* utilizing the *j*-th Monte-Carlo simulation. Let $∥E_{k}^{j}-E_{k}∥$ indicate the target position estimate error at sample-step *k*. For representing the position estimate error, one uses

(33) $$\begin{array}{c} RMSE_{k}=\sqrt{\frac{\sum_{j=1}^{M_{c}}{∥E_{k}^{j}-E_{k}∥}^{2}}{M_{c}}}. \end{array}$$

The computational load is an evaluation indicator of algorithms. Let CompLoad denote the computational time for all MC simulations under MATLAB simulations.

### 4.1. Scenario 1

Scenario 1 is as follows. Initially, the ownship is positioned at (0, 0, 100) in meters. The ownship moves with a constant velocity (5, 0, 0) in m/s for 1000 sample-steps. From sample-step 1000 to 1150, the ownship performs the coordinates turn (CT) in the xy-plane with turn rate ω=2 degrees per second. Here, the ownship’s CT is modeled using

(34) $$\begin{array}{c} X_{k+1}^{O}=F^{CT}X_{k}^{O}. \end{array}$$

Here, $F^{CT}$ is

(35) $$\begin{array}{c} F^{CT}=\left(\begin{array}{cccccc} 1 & 0 & 0 & dt\times S_{w} & -dt\times C_{w} & 0\\ 0 & 1 & 0 & dt\times C_{w} & dt\times S_{w} & 0\\ 0 & 0 & 1 & 0 & 0 & dt\\ 0 & 0 & 0 & c(\omega \times dt) & -s(\omega \times dt) & 0\\ 0 & 0 & 0 & s(\omega \times dt) & c(\omega \times dt) & 0\\ 0 & 0 & 0 & 0 & 0 & 1 \end{array}\right). \end{array}$$

Here, $S_{w}=\frac{s(\omega \times dt)}{\omega \times dt}$, and $C_{w}=\frac{1-c(\omega \times dt)}{\omega \times dt}$.

Initially, the target is positioned at (2000, 0, 0) in meters. We consider a constant-velocity target, whose speed is set as Sp=2 m/s. In (4), the target’s velocity vector is set as

(36) $$\begin{array}{c} \left({\dot{x}}_{k}^{E},{\dot{y}}_{k}^{E},{\dot{z}}_{k}^{E}\right)=\left(Sp\times c\left(\theta \right)\times s\left(\psi \right),Sp\times c\left(\theta \right)\times c\left(\psi \right),Sp\times s\left(\theta \right)\right). \end{array}$$

Here, θ=π, and $\psi =\frac{3\pi }{4}$ is used.

**Figure 1:**
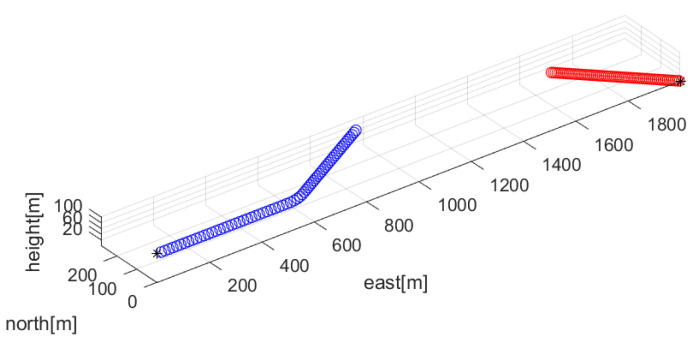
Scenario 1. The target’s trajectory is shown as red circles. In addition, the ownship’s trajectory is shown as blue circles. A black asterisk denotes the initial position of the target or the ownship.

Figure 1 depicts Scenario 1. In this figure, the target’s trajectory is shown as red circles. In addition, the ownship’s trajectory is shown as blue circles. A black asterisk denotes the initial position of the target or the ownship.

**Figure 2:**
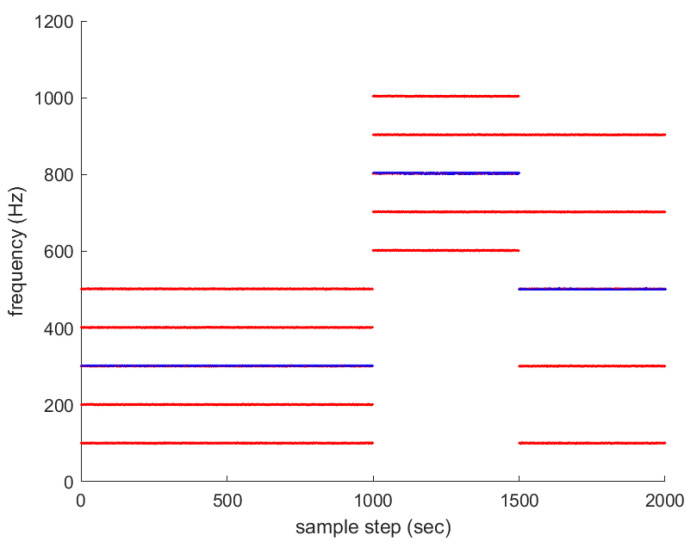
Associated with Scenario 1 in Figure 1, multiple frequency lines $f_{k}^{m}$ ($m\in \{1,2,\ldots ,M_{k}\}$) are depicted in red. The average frequency lines $f_{k}^{avg}$ are depicted in blue. The frequency lines disappear and appear occasionally. All new frequency lines appear at 1000 sample-steps, while the existing frequency lines disappear at the moment.

Associated with Scenario 1 in Figure 1, multiple frequency lines $f_{k}^{m}$ ($m\in \{1,2,\ldots ,M_{k}\}$) are depicted with red color in Figure 2. The average frequency lines $f_{k}^{avg}$ are depicted in blue. The frequency lines disappear and appear occasionally. All new frequency lines appear at 1000 sample-steps, while the existing frequency lines disappear at the moment.

**Figure 3:**
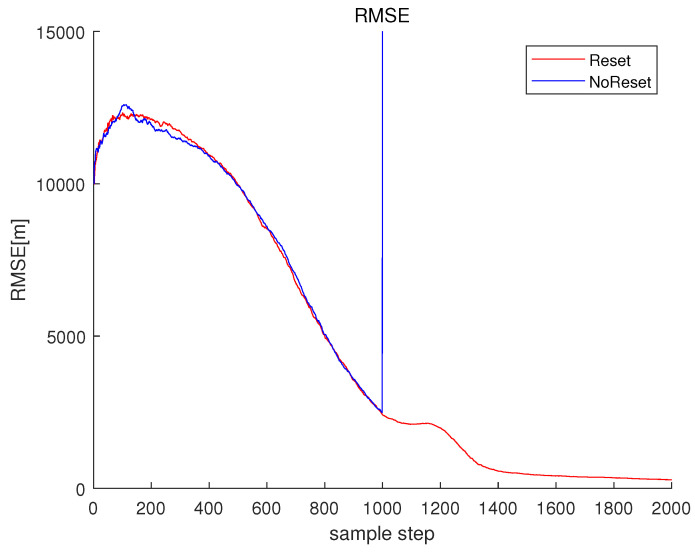
RMSE of Scenario 1. Reset depicts $RMSE_{k}$ (33) under the proposed filter reset strategy in Section 3.2. Under the proposed Reset, $RMSE_{k}$ converges to almost zero as time goes on. NoReset depicts $RMSE_{k}$ (33) in the case where the filter reset strategy in Section 3.2 is not applied.

Associated with Scenario 1 in Figure 1, Reset in Figure 3 depicts $RMSE_{k}$ (33) under the proposed filter reset strategy in Section 3.2. For Reset, CompLoad is 31 s. Since each scenario lasts for 2000 s, the proposed filter is suitable for real-time applications. As time goes on, $RMSE_{k}$ under Reset converges to almost zero.

For comparison, one simulates the case where one does not reset the EKF estimate. In other words, one simulates the case where the filter reset strategy in Section 3.2 is not applied. Associated with Scenario 1 in Figure 1, NoReset in Figure 3 depicts $RMSE_{k}$ (33) in the case where the filter reset strategy in Section 3.2 is not applied. The RMSE suddenly increases at 1000 s, since frequency lines change at this moment. For NoRest, CompLoad is 33 s.

**Figure 4:**
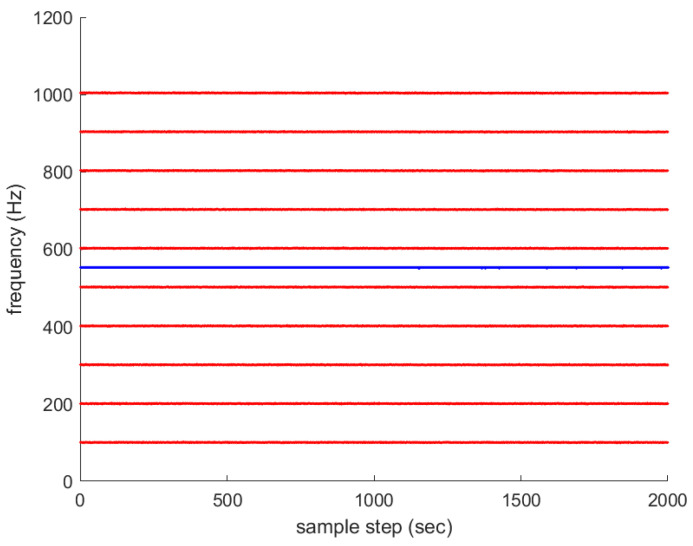
Associated with Scenario 1 in Figure 1, multiple frequency lines $f_{k}^{m}$ ($m\in \{1,2,\ldots ,M_{k}\}$) are depicted in red. The average frequency line $f_{k}^{avg}$ is depicted in blue. No frequency lines disappear in this ideal scenario.

For another comparison, one simulates the ideal case where all frequency lines exist at all sample-steps. Associated with Scenario 1 in Figure 1, multiple frequency lines $f_{k}^{m}$ ($m\in \{1,2,\ldots ,M_{k}\}$) are depicted with red color in Figure 4. The average frequency line $f_{k}^{avg}$ is depicted in blue. No frequency lines disappear in this scenario.

**Figure 5:**
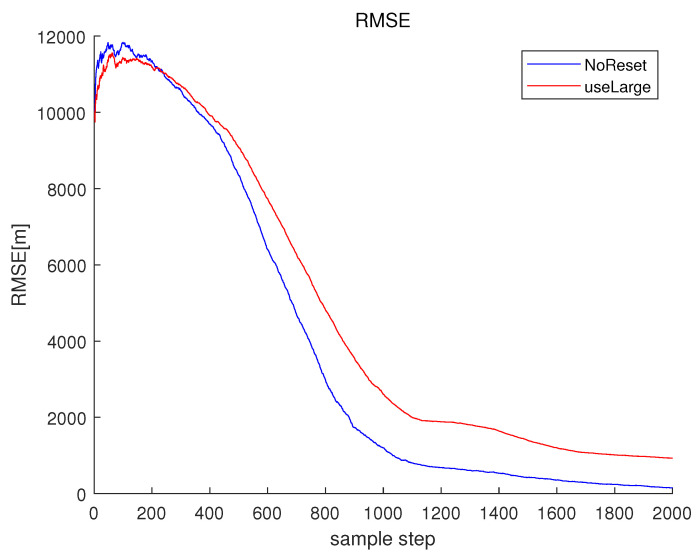
RMSE of the scenario in Figure 4. NoReset depicts $RMSE_{k}$ in the case where the filter reset strategy in Section 3.2 is not applied. In NoReset, we use the average frequency line $f_{k}^{avg}$ as our filter state. In this figure, useLarge depicts $RMSE_{k}$ as we track every frequency line one by one.

Associated with the scenario in Figure 4, NoReset in Figure 5 depicts $RMSE_{k}$ in the case where the filter reset strategy in Section 3.2 is not applied. In NoReset, we use the average frequency line $f_{k}^{avg}$ as our filter state. Considering NoReset, CompLoad is 34 s.

Using (12) as the measurement equation, we can build a 3D AFTMA filter for tracking every frequency line one by one. For tracking every frequency line one by one, we initialize the EKF as follows. Since there are *M* frequency lines in total, ${\hat{X}}_{0|0}^{M}$ is initialized as

(37) $$\begin{array}{c} {\hat{X}}_{0|0}^{M}=\left(\begin{array}{c} \left(d_{0}+d_{e}\right)\times c\left(b_{2,0}\right)\times c\left(b_{1,0}\right)\\ \left(d_{0}+d_{e}\right)\times c\left(b_{2,0}\right)\times s\left(b_{1,0}\right)\\ \left(d_{0}+d_{e}\right)\times s\left(b_{2,0}\right)\\ 0_{3,1}\\ f_{0}^{1}\\ f_{0}^{2}\\ \cdots \\ f_{0}^{M} \end{array}\right). \end{array}$$

Here, *M* is used to indicate that *M* frequency lines are used in the KF estimate. The covariance matrix $P_{k|k}^{M}$ has size (6+M)×(6+M). The covariance matrix’s submatrix $P_{0|0}^{M}\left[1:6,1:6\right]$ is initialized using $P_{0|0}\left[1:6,1:6\right]$ in (31). Moreover, the error covariance for each frequency line $f_{0}^{j}$ (j∈{1,2,…,M}) is initialized as ${\left(\delta f^{j}\right)}^{2}/3$. Here, $\delta f^{j}=\frac{f_{0}^{j}\times V_{max}}{C}$.

Associated with the scenario in Figure 4, useLarge in Figure 5 depicts $RMSE_{k}$ as we track every frequency line one by one. Considering useLarge, CompLoad is 64 s. We track every frequency line one by one, and the computational load increases compared to the case where we use the average frequency line $f_{k}^{avg}$ as our filter state. Moreover, the RMSE increases compared to the case where we use the average frequency line as our filter state. Note that using the average frequency as our filter state, we can decrease the matrix size of the covariance matrix in the Kalman filter. This decreases the numerical error in the matrix calculations, such as the calculation of an inverse matrix.

### 4.2. Scenario 2

**Figure 6:**
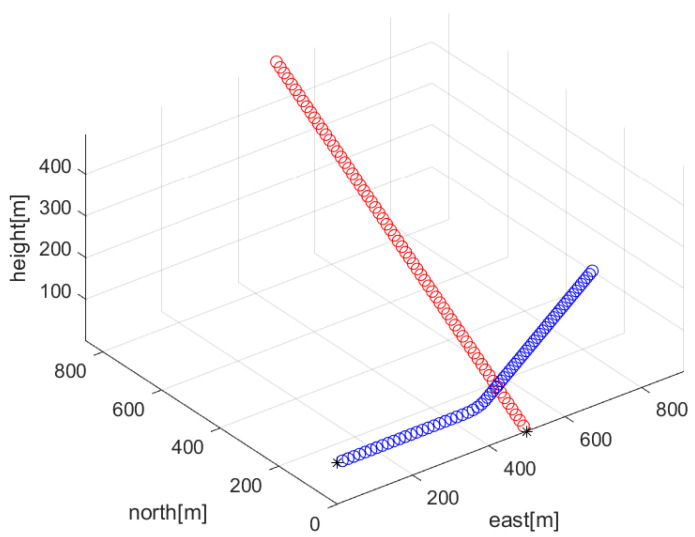
Scenario 2. The target’s trajectory is shown as red circles. In addition, the ownship’s trajectory is shown as blue circles. A black asterisk denotes the initial position of the target or the ownship.

Figure 6 depicts Scenario 2. In this scenario, the trajectory of the ownship is identical to that in Scenario 1. A constant-velocity target starts from (500,0,0), and it moves with speed Sp=5 m/s. In (4), the target’s velocity vector is set as

(38) $$\begin{array}{c} \left({\dot{x}}_{k}^{E},{\dot{y}}_{k}^{E},{\dot{z}}_{k}^{E}\right)=\left(Sp\times c\left(\theta \right)\times s\left(\psi \right),Sp\times c\left(\theta \right)\times c\left(\psi \right),Sp\times s\left(\theta \right)\right). \end{array}$$

Here, $\theta =\frac{\pi }{6}$, and $\psi =\frac{3\pi }{4}$ is used.

In Figure 6, the target’s trajectory is shown as red circles. In addition, the ownship’s trajectory is shown as blue circles. A black asterisk denotes the initial position of the target or the ownship.

**Figure 7:**
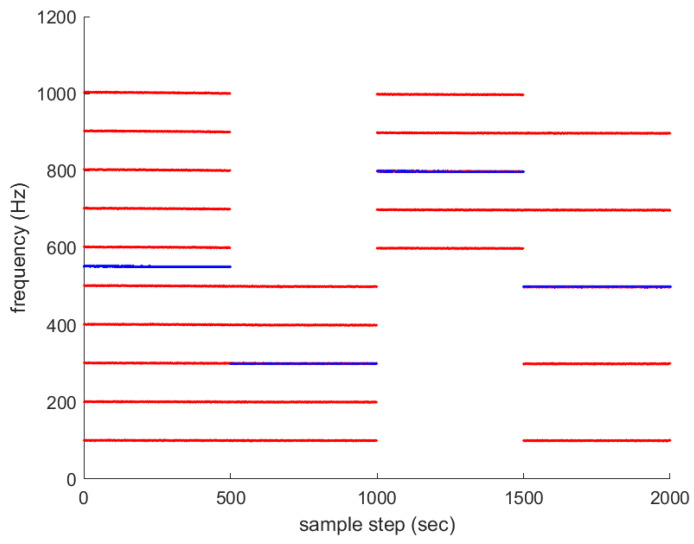
Associated with Scenario 2 in Figure 6, multiple frequency lines $f_{k}^{m}$ ($m\in \{1,2,\ldots ,M_{k}\}$) are depicted in red. The average frequency lines $f_{k}^{avg}$ are depicted in blue. The frequency lines disappear and appear occasionally.

Associated with Scenario 2 in Figure 6, multiple frequency lines $f_{k}^{m}$ ($m\in \{1,2,\ldots ,M_{k}\}$) are depicted with red color in Figure 7. The average frequency lines $f_{k}^{avg}$ are depicted in blue. The frequency lines disappear and appear occasionally.

**Figure 8:**
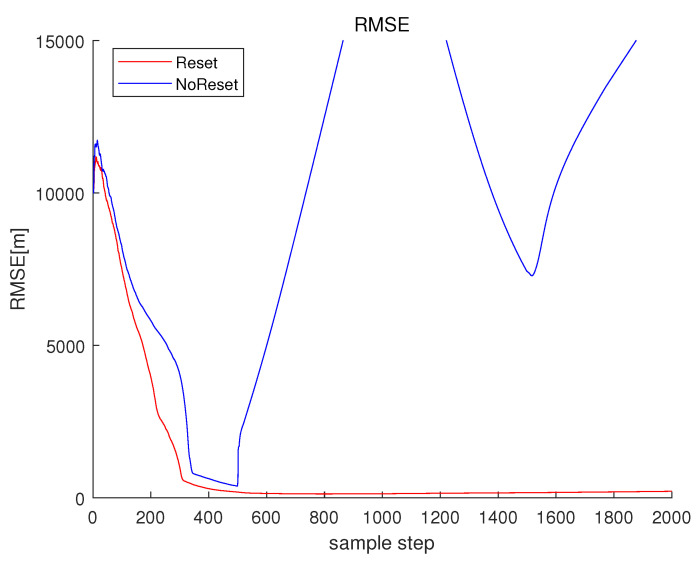
RMSE of Scenario 2. Reset depicts $RMSE_{k}$ (33) under the proposed filter reset strategy in Section 3.2. NoReset depicts $RMSE_{k}$ in the case where the filter reset strategy is not applied.

Associated with Scenario 2 in Figure 6, Reset in Figure 8 depicts $RMSE_{k}$ (33) under the proposed filter reset strategy in Section 3.2. As time goes on, $RMSE_{k}$ under Reset converges to almost zero. CompLoad under Reset is 33 s. The proposed tracking filter is suitable for real-time applications.

For comparison, one simulates the case where the filter reset strategy in Section 3.2 is not applied. Associated with Scenario 2 in Figure 6, NoReset in Figure 8 depicts $RMSE_{k}$ in the case where the filter reset strategy in Section 3.2 is not applied. The RMSE of NoReset suddenly increases at 500 s, since frequency lines change at this moment (see Figure 7). Considering NoReset, CompLoad is 34 s.

**Figure 9:**
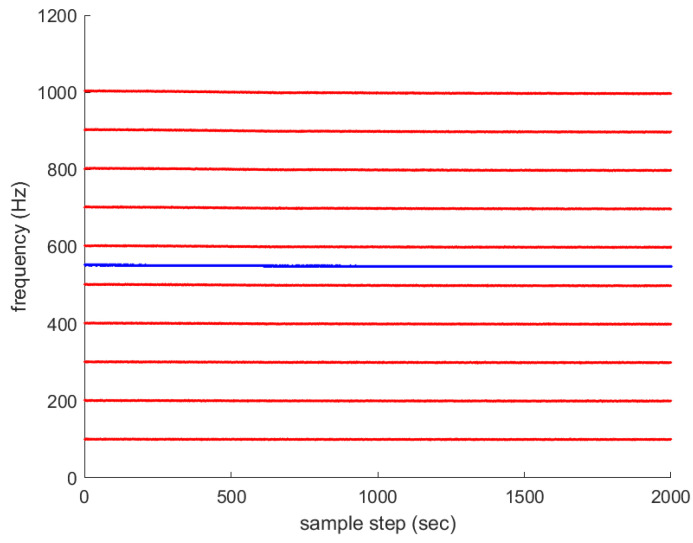
Associated with Scenario 2 in Figure 6, multiple frequency lines $f_{k}^{m}$ ($m\in \{1,2,\ldots ,M_{k}\}$) are depicted in red. The average frequency line $f_{k}^{avg}$ is depicted in blue. No frequency lines disappear in this ideal scenario.

For another comparison, one simulates the ideal case where all frequency lines exist at all sample-steps. Associated with Scenario 2 in Figure 6, multiple frequency lines $f_{k}^{m}$ ($m\in \{1,2,\ldots ,M_{k}\}$) are depicted with red color in Figure 9. The average frequency line $f_{k}^{avg}$ is depicted in blue. No frequency lines disappear in this scenario.

**Figure 10:**
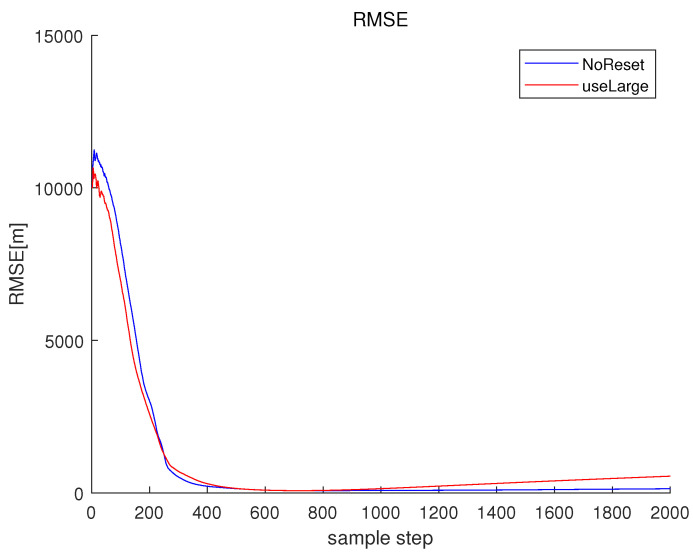
RMSE of the scenario in Figure 9. NoReset depicts $RMSE_{k}$ in the case where the filter reset strategy in Section 3.2 is not applied. In NoReset, we use the average frequency line $f_{k}^{avg}$ as our filter state. In this figure, useLarge depicts $RMSE_{k}$ as we track every frequency line one by one.

Associated with the scenario in Figure 9, NoReset in Figure 10 depicts $RMSE_{k}$ in the case where the filter reset strategy in Section 3.2 is not applied. We use the average frequency line $f_{k}^{avg}$ as our filter state. Under NoReset, CompLoad is 42 s.

Using (12) as the measurement equation, we can build a 3D AFTMA filter for tracking every frequency line one by one. For tracking every frequency line one by one, we initialize the EKF state vector ${\hat{X}}_{0|0}^{M}$ using (37).

Associated with the scenario in Figure 9, useLarge in Figure 10 depicts $RMSE_{k}$ as we track every frequency line one by one. CompLoad under useLarge is 65 s. Since we track every frequency line one by one, the computational load increases compared to the case where we use the average frequency line as our filter state. Moreover, the RMSE of useLarge increases compared to the case where we use the average frequency line as our filter state.

## 5. Conclusions

In the 3D AFTMA problem, the ownship tracks a 3D target based on frequency, elevation, and azimuth measurements. This study addresses 3D AFTMA problems so that the ownship can track a target while measuring the target’s sound with multiple frequency lines. Instead of tracking every frequency line one by one, we propose to estimate the average emitting frequency by setting the average frequency as the state vector in the filter. Moreover, our tracking filter handles the case where some frequency lines may disappear and appear occasionally. MATLAB simulations demonstrate the performance of the proposed tracking approaches. In the future, we will perform experiments utilizing a real ownship platform to demonstrate the proposed tracking approaches.

## Data Availability

Not applicable.
